# Sympathetic Hyperactivity and Age Affect Segregation and Expression of Neurotransmitters

**DOI:** 10.3389/fncel.2018.00411

**Published:** 2018-11-13

**Authors:** Candelaria Merino-Jiménez, Filiberto Miguel, Jessica Abigail Feria Pliego, María Elena Zetina Rosales, Fredy Cifuentes, Miguel Angel Morales

**Affiliations:** Departamento de Biología Celular & Fisiología, Instituto de Investigaciones Biomédicas, Universidad Nacional Autónoma de México, Ciudad de México, Mexico

**Keywords:** co-transmission, sympathetic ganglia, stress, hypertension, SHR, acetylcholine, GABA

## Abstract

Sympathetic neurons of the rat superior cervical ganglion (SCG) can segregate their neurotransmitters and co-transmitters to separate varicosities of single axons. We have shown that transmitter segregation is a plastic phenomenon and that it is correlated with the strength of synaptic transmission. Here, we determined whether sympathetic dysfunction occurring in stress and hypertension was correlated with plastic changes of neurotransmitter segregation. We characterized the expression of the markers, L-glutamic acid decarboxylase of 67 kDa (GAD67) and vesicular acetylcholine (ACh) transporter (VAChT) in the SCG of cold stressed and spontaneously hypertensive rats (SHR). Considering that the SCG comprises a heterogeneous neuronal population, we explored whether the expression and segregation of neurotransmitters would also have an intraganglionic heterogeneous distribution in ganglia of stressed and hypertensive rats. Furthermore, since hypertension in SHR is detected around 8–10 weeks, we evaluated expression and segregation of ACh and GABA in adult hypertensive (12-week old (wo)) and young pre-hypertensive (6-wo) SHR. We found an increase in segregation of ACh and GABA with no change in transmitter expression in ganglia of stressed animals. In contrast, in SHR, there was an increase in GABA expression, although segregation did not vary. Segregation showed a caudo-rostral gradient in controls but not in the ganglia of stressed animals. GABA expression showed a rostro-caudal gradient in adult SHR, which was not present in young 6-wo rats. In young SHR, ACh increased and, unexpectedly, segregation of ACh and GABA was higher than in adults. Data suggest that ACh and GABA segregation increases in acute sympathetic hyperactivity like stress, but does not vary in chronic hyperactivity such as in hypertension. Changes in segregation are age-dependent and might be involved in the mechanisms underlying stress and hypertension.

## Introduction

The sympathetic nervous system (SNS) regulates many functions including blood pressure, cardiac contractility, intestinal motility and exocrine gland secretion, among others. To achieve these functions, the SNS requires a proper balance in content and cellular distribution of its neurotransmitters. It is known that peripheral efferent sympathetic activity is sustained by the effect of a main transmitter, acetylcholine (ACh) at preganglionic and norepinephrine (NE) at postganglionic level, and by the action of two or more concurrently released co-transmitters. This property of releasing more than one transmitter it is known as neuronal co-release and allows neurons to use more than one neurotransmitter to convey signals across the synaptic cleft (Burnstock, 1976; Kupfermann, 1991). Initially, it was believed that co-release was carried out by the co-storage of the same set of neurotransmitters at all presynaptic endings (Chan-Palay and Palay, 1984; Burnstock, 1990). However, later evidence showed that neurons are able to segregate their neurotransmitters, and independently store and release them at distinct terminals, allowing neurons to exert each transmitter function separately (Fisher et al., 1988; Hattori et al., 1991; Morales et al., 1995; Sámano et al., 2012; Zhang et al., 2015). Like other neurons, sympathetic ones have this capability to segregate their neurotransmitters (Morales et al., 1995; Chanthaphavong et al., 2003; Sámano et al., 2006, 2009; Vega et al., 2010, 2016).

Neurotransmitter segregation is a plastic phenomenon that depends on neuronal requirements (Sámano et al., 2012). For instance, we demonstrated that in rat sympathetic neurons, both *in vitro* and *in vivo*, neurotransmitter segregation is modulated by neurotrophic factors (Vega et al., 2010, 2016). Additionally, we found a likely functional role of segregation in the rat superior cervical ganglia (SCG), we detected a correlation between the level of segregation of ACh and GABA, and the strength of synaptic transmission, where more segregation correlates with stronger synaptic transmission (Elinos et al., 2016). Considering this evidence regarding the plasticity of neurotransmitter segregation, and its likely functional roles, here we explored whether certain physio-pathologic conditions coursing with sympathetic dysfunction, like stress and hypertension, also correlate with changes in the segregation of neurotransmitters in the rat SCG.

It has been reported that in stress and hypertension there is an increase in sympathetic activity (Guyenet, 2006; Lambert and Lambert, 2011). Stress is defined as a state of disharmony, or threatened homeostasis (Chrousos and Gold, 1992). It is worth to note that stress is not always noxious, stress can be also associated to eustress, i.e., a condition perceived as pleasant or exciting, that could be positive stimulus to emotional and intellectual growth and development (Selye, 1950). Stress increases the rate and force of cardiac contraction, respiratory frequency, metabolism and blood flow, in response to an increase in sympathetic nervous activity (Jansen et al., 1995; Kvetnansky et al., 2013). It is known that different stressors induce different patterns of activation of the SNS (Ibrahim et al., 2015). Thus, stress in addition to altering the synthesis of catecholamines in the sympato-adrenomedular system, affects the function of the sympato-neural system, which innervates most of the organs (Goldstein and Kopin, 2008; Lambert and Lambert, 2011; Ibrahim et al., 2015). Sympathetic ganglia like other structures of the sympato-neural system undergo changes in stress conditions; for example, immobilization stress elevates tyrosine hydroxylase (TH) and neuropeptide Y (NPY) mRNAs in rat SCG (Nankova et al., 1996); and stress induced by exposure to a cold environment increases TH activity in sympathetic ganglia (Ulus and Wurtman, 1979; Kvetnansky and Sabban, 1993).

Enhanced sympathetic nerve activity has been detected and implicated in the pathophysiology of hypertension, either in animal models and hypertensive patients (Lundin and Thorén, 1982; Magee and Schofield, 1992, 1994; Mancia et al., 1999; Guyenet, 2006). For instance, it has been shown that essential-hypertensive patients display plasma NE values greater than those of normotensive individuals (Mancia et al., 1999). Furthermore, microneurographic approaches showed that sympathetic nerve traffic increases progressively from the normotensive to the moderately and more severe essential-hypertensive state (Schlaich et al., 2004).

We hypothesized that in stress and hypertension, SNS overactivity might change the distribution of ganglionic neurotransmitters in the SCG of the rat, to cope with sympathetic dysfunction. To explore this hypothesis, we investigated the expression and segregation of ACh and GABA, by immunostaining of vesicular ACh transporter (VAChT) and L-glutamic acid decarboxylase of 67 kDa (GAD67), in animals subjected to cold stress, and in spontaneously hypertensive rats (SHR). Considering the presence of sympathetic overactivity, we evaluated GABA, an inhibitory neurotransmitter, whose level of expression could have changed to counteract the sympathetic hyperfunction. The neuronal population of the rat SCG is diverse, and can be grouped into rostral and caudal based on their regional distribution (Dail and Barton, 1983; Flett and Bell, 1991; Elinos et al., 2016). Since the rostral and caudal neurons might be differentially involved in sympathetic overactivity, we asked whether changes in expression and segregation of ACh and GABA could be different in each ganglionic region. Finally, considering that in SHR, hypertension is detected around the age of 8–10 weeks (Lee et al., 1991; Li et al., 2012), we determined the expression and segregation of sympathetic transmitters at two ages, in pre-hypertensive young 6-week old (wo) and in hypertensive adult 12-wo SHR. We found an increase in the segregation of ACh and GABA under stress conditions, with no changes in the level expression of these neurotransmitters, while in hypertension, segregation did not vary, but GABA expression increase in SHR at both ages. Unexpectedly, we found greater segregation and higher GABA and ACh content in young SHR and control normotensive Wistar Kyoto (WKy) rats, compared to adults.

## Materials and Methods

### Animals

Experimental procedures were conducted according to the ethical guidelines for the use of laboratory animals of the National Academy of Sciences of the United Sates and approved by our Institutional Committee for the Care and Use of Animals in the Laboratory. To study the distribution of ganglionic neurotransmitters in the SCG of the rat in response to hypertension we used SHR adult and young male rats, and as a control normotensive rats of the same strain, the WKy. For the stress model, we use male Wistar rats between 8- and 9-wo (240–260 g), because all our previous data in segregation and expression of ACh and GABA have been observed in this strain.

### Stress Induction by Prolonged Exposure to Cold

Male Wistar rats were divided into two groups. One group was subjected to stress by keeping them at a temperature of 5°C for 24 h during a period of 5 days under controlled conditions of 12 h light-dark cycles with food and water *ad libitum*. The second group was used as the control. The SCG and spinal cord were dissected out immediately after stress induction. The stress induction by cold was confirmed by measuring body weight and the expression level of TH protein and mRNA in the adrenal medulla (AM).

### RNA Extraction and RT-PCR Analyses

For this assay, we used 11 stressed and eight control rats. After stress induction, the AM was removed and immediately frozen in liquid nitrogen. In each animal, total RNA was isolated using Trizol following the manufacturer’s instructions (Invitrogen, Life Technologies). Total RNA (1.5 μg) was reverse transcribed using SuperScript II reverse transcriptase (Life Technologies) and 500 ng of random primers (Promega, Madison, WI, USA). For TH mRNA 2 μl of complementary DNA (cDNA) was amplified by PCR Master Mix (Promega) using oligonucleotides designed to amplify a fragment of 646 bp (nt 720–1365) from TH, F (5′GAAGGGCCTCTATGCTACCCA) and R (5′TGGGCGCTGGATACGAGA) based on the TH mRNA of *Rattus norvegicus* (NM 012740.3). For actin mRNA analyses, oligonucleotides designed to amplify a fragment of 236 bp (nt 451–686) from actin, F (5′GAGACCTTCAACACCCC) and R (5′GTGGTGGTGAAGCTGTAGCC) based on the actin mRNA of *Rattus norvegicus* (NM 031144.3). The cycle conditions used for both fragments were: 20 cycles at 94°C for 30 s, 63°C for 30 s and 72°C for 30 s. Amplified products were analyzed by electrophoresis in agarose gels pre-stained with ethidium bromide. The gels were digitalized using Typhoon FLA 9500 scanner (GE Healthcare Bio-Sciences Corp., Piscataway, NJ, USA). Band intensity was analyzed using ImageJ software (US National Institutes of Health).

### Western Blot Assay

For protein assay we used five stressed and four control rats. After stress induction, the AM was removed and homogenized in lysis buffer (in mM: 150 NaCl, 1 EDTA, 0.5 DTT, 20 Tris, 0.5% Triton X-100, pH 8.0) containing 1% SDS and 1× proteinase inhibitor cocktail (Roche Applied Sciences, Indianapolis, IN, USA). Protein concentration was determined by Bradford method. Then, 15 μg of protein were resolved on 12.5% polyacrylamide-SDS gels and transferred onto PVDF (Immobilon-P, Millipore, Billerica, MA, USA) membranes. The membranes were blocked with 5% non-fat dry milk for 2 h and then incubated overnight at 4°C with mouse anti-TH polyclonal antibody (Millipore; 1:15,000) and goat anti-actin polyclonal antibody (Santa Cruz; 1:1,000). Immunoreactive bands were detected with anti-mouse (Millipore; 1:10,000) and anti-goat (Bethyl; 1:10,000) horseradish peroxidase-conjugated secondary antibodies, using Immobilon Western Chemiluminescent HRP substrate (Millipore). Antibody dilutions and rinses were carried out in PBS-Tween 5%.

### Histological Procedures for Decentralization of SCG and Retrograde Labeling of Sympathetic Preganglionic Neurons

In a set of rats, we decentralized one of the SCG by transecting the sympathetic cervical trunk (SCT) 3–5 mm caudal to the ganglion. Seven days later the ganglion was dissected, removed and processed for immunocytochemistry. To retrograde labeling and immunostaining cell bodies of SPN, in other groups of rats, the SCT of one side was cut 10–12 mm from the spinal cord (SC) and the Fluoro-Gold (FG) tracer (Fluorochrome, LLC, Denver, CO, USA) was applied at the distal end. Three days later SC was dissected, removed and processed for immunolabeling. Rats were anesthetized with sodium pentobarbital (125 mg/kg i.p.), and transcardiacally perfused with 100 ml of ice-cold phosphate buffered saline (0.01 M PBS, pH 7.4) for 3 min and then with 250 ml of ice-cold fixative solution (2% paraformaldehyde, 0.18% picric acid in 0.1 M PBS, pH 7.4), 100 ml for 3 min and the remaining 150 ml for 40 min. One of the SCG and a SC segment (from C8 to T3) were quickly dissected and postfixed overnight in the same fixative solution and cryoprotected in sucrose solution (10%–30% w/v). Longitudinal sections of SCG and horizontal or transverse sections of SC were cut at 12 μm thickness on a cryostat (LEICA CM1520) at −20°C and recovered on Superfrost plus gelatin-coated slides (Electron Microscopy Sciences, Halfied, PA, USA). For ganglia, longitudinal slices were randomly sampled from 40 to 45 sections cut along its z-axis, while for SC, slices containing the intermediolateral nuclei were processed for double immunolabeling.

### Immunocytochemistry Assays

The tissue sections were washed once with PBS, permeabilized and blocked with 10% bovine serum albumin (BSA), 0.3% Triton X-100 for 2 h. Primary antibodies, goat polyclonal anti-choline acetyl transferase (ChAT, the enzyme responsible for the synthesis of ACh) and anti-VAChT, and mouse monoclonal anti-L-GAD67 (the enzyme responsible for the synthesis of GABA), were incubated overnight in a humid atmosphere at room temperature. Specific conditions used for each antibody are given in Table 1. Tissue sections were washed twice for 15 min each in 0.1 M PBS, 0.3% Triton X-100 and then incubated for 2 h with the appropriate secondary antibody (Table 1). After incubation the tissue sections were washed again and finally mounted on slides with fluorescence mounting medium (Dako, Santa Clara, CA, USA).

**Table 1:** Antibodies used for immunohistochemistry.

Antiserum	Type of antibody	Conjugate to	Dilution	Source	Catalogue number
Primary					
ChAT (human)	Goat polyclonal		1:200 (IF)	Millipore, Chemicon, MA, USA	AB144P
VAChT (rat)	Goat polyclonal		1:400 (IF)	Immunostar, Inc.,	24286
GAD67 (synthetic)	Mouse polyclonal		1:200 (IF)	Millipore, Chemicon, MA, USA	MAB5406
TH (rat)	Mouse polyclonal		1:7,500 (WB)	Millipore, Chemicon, MA, USA	MAB318
Secondary					
α goat IgG	Donkey	Alexa 488	1:1,000 (IF)	Jackson ImmunoResearch Lab, Inc., ME, USA	705-545-147
α mouse IgG	Donkey	Alexa 594	1:750 (IF)	Jackson ImmunoResearch Lab, Inc., ME, USA.	715-585-150

### Image Acquisition and Analysis

From a selected slice, immunofluorescence tile images (35–40, depending on the size of the section) were sequentially acquired with a Nikon A1R+ laser scanning confocal head coupled to an Eclipse Ti-E inverted microscope (Nikon Corporation, Tokyo, Japan) equipped with a XYZ motorized stage (TI-S-ER, Nikon). Tile imaging was done with a CFI Plan Apo lambda 40× (N.A. 0.95), using 1.4 mW of 488 nm and 0.7 mW of 561 nm laser power, pinhole aperture of 38.31 μm, GaAsP detectors and image stitching set at 17% overlapping, all controlled through NIS Elements C software v5.00 (Nikon).

Using the Metamorph image analysis system (v. 7.5.6; Universal Imaging Corporation, Molecular Devices, Downingtown, PA, USA), we removed out of focus blur by means of deconvolution functions. We then identified the specific labels by selecting puncta optical density (OD) that surpassed the negative staining background level (i.e., puncta OD > background mean + 2 SD). We assessed the number of overlapping pixels for each marker in double-labeled varicosities in the complete ganglion section and their regions (rostral and caudal). The area occupied by VAChT and GAD67 was expressed as a percentage of the whole section area. The co-occurrence of the two labels was expressed as the ratio of the percentage of fibers co-expressing the two labels relative to the percentage of fibers expressing only one of them. For the SPN cells, we counted the number of all immnunostained cell bodies and determined the percent of GAD67 immunoreactive cells that co-stain for ChAT.

### Blood Pressure Measurement

Systolic blood pressure (SBP) was measured prior to experiments with an indirect tail-cuff apparatus. Adult SHR had SBP over 160 mm Hg, while control WKy rats and young 6-wo SHR had SBP <120 mm Hg.

### Statistics

Results obtained from independent experiments were expressed as mean ± SEM. The differences between pairs of groups of different experimental conditions were evaluated with an unpaired Student’s *t*-test. For analyzing segregation and expression of neurotransmitters along the ganglionic regions, we employed one-way ANOVA using Tukey’s multiple comparison test. To evaluate if age is an important factor that changes the expression and distribution of GAD67 and VAChT in hypertension, we performed two-way ANOVA followed by Bonferroni post-tests. *P*-values < 0.05 were considered statistically significant for the quantification of immunofluorescence density and segregation values.

## Results

### Stress Model

#### Cold-Stress Enhanced Segregation of ACh-GABA in SCG

**Figure 1:**
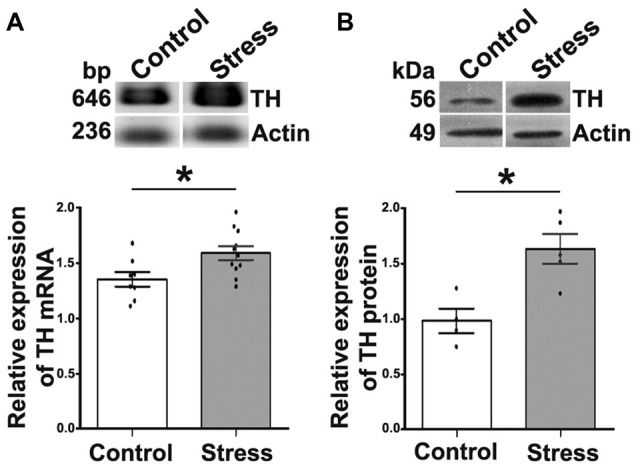
Cold-stress increased expression of tyrosine hydroxylase (TH) mRNA and protein in adrenal medulla (AM). **(A)** Conventional RT-PCR analysis performed on total RNA obtained from AM showing an increase (17.5%) in TH mRNA after 5 days of cold-stress. RT-PCR assay was performed for the amplification of TH mRNA fragment of 646 bp. **(B)** Western blot (WB) analysis of TH protein (56 kDa) expression showing an increase (1.6-fold) after cold-stress. Graphs show quantification of TH relative to actin, mean ± SEM and the significance level. Actin was used as the housekeeping gene for RT-PCR and as the loading control for WB assays. ^*^P < 0.05.

To assess the presence of stress and consequently of sympathetic hyperactivity in cold exposed rats, we determined body weight and the expression of TH in the AM. Rats exposed to cold despite they consumed the same amount of food as control rats, lost 5%–10% of their weight (10.0 ± 2.3 g) in contrast to control rats that gained 22.0 ± 3.3 g (*P* < 0.05) over the same period. In agreement with previous reports (Ulus and Wurtman, 1979; Kvetnansky and Sabban, 1993; Liu et al., 2005), we found that cold stress elevated significantly both TH mRNA and TH content in rat AM; TH mRNA increased 17.6% and TH protein 1.6-fold (*P* < 0.05; Figure 1).

**Figure 2:**
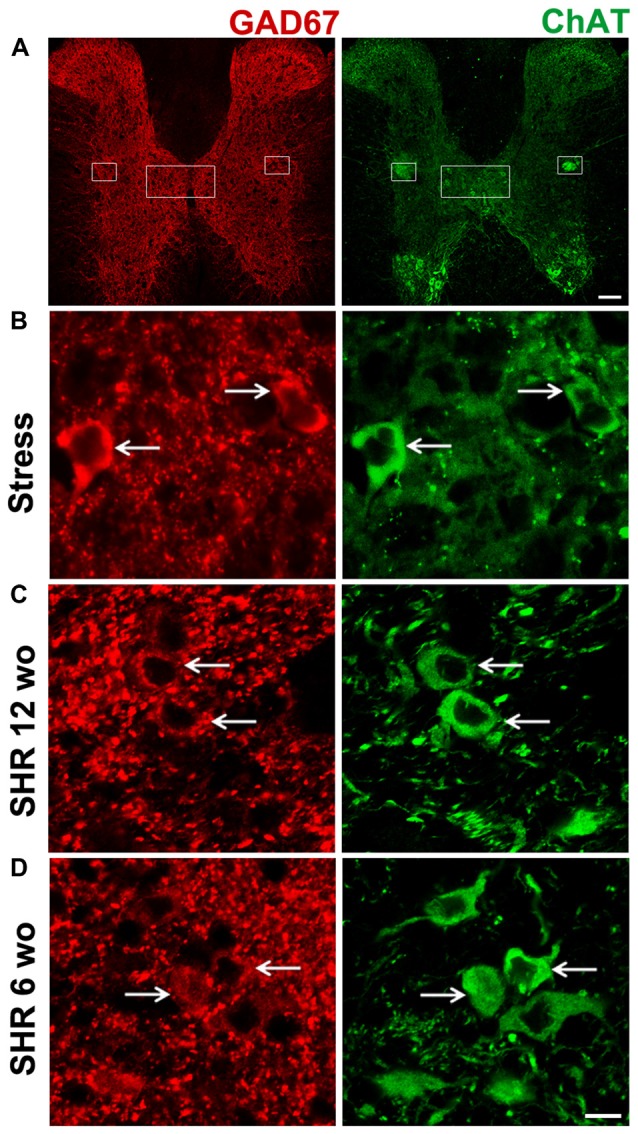
All sympathetic preganglionic neuronal cell bodies positive for L-glutamic acid decarboxylase of 67 kDa (GAD67), co-expressed ChAT. Double immunostaining in sections of spinal cord for GAD67 (red) and ChAT (green). **(A)** Low magnification of transverse section of spinal cord depicting the whole gray region, the sympathetic nuclei are shown with boxes. **(B)** Micrograph of transverse section of spinal cord in cold-stressed rats (*n* = 4). **(C)** Micrograph of transverse section of spinal cord in spontaneously hypertensive rats (SHR) adult 12-week old (wo) rats (*n* = 4). **(D)** Micrograph of longitudinal section of spinal cord in SHR young 6-wo rats (*n* = 3). GAD67-IR cell bodies co-expressing ChAT are shown with arrows. Scale bar = 100 μm **(A)**, 10 μm **(B–D)**.

**Figure 3:**
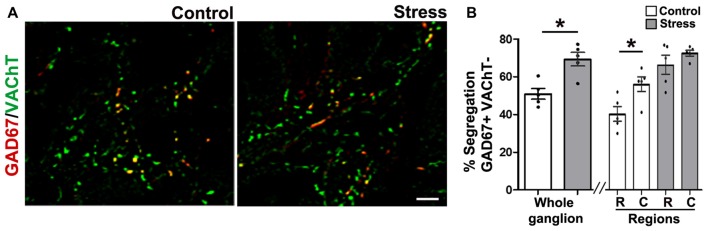
Cold-stress increased segregation of acetylcholine (ACh) and GAD67. **(A)** Merged images showing the immunolabeling of GAD67 (red), vesicular ACh transporter (VAChT; green) and co-occurrence of both labels (yellow) in superior cervical ganglion (SCG) from control and cold-stressed rats. **(B)** Graphs showing percent segregation of VAChT/GAD67 in SCG of stressed rats with respect to control. Stress increased the percentage of segregation in the whole ganglion; 67.8 ± 4.4% compared to 50.1 ± 3.6% in control rats (*P* < 0.05). The caudo-rostral ganglionic regionalization of VAChT/GAD67 segregation found in control animals (rostral 37.1 ± 3.4%, caudal 55.4 ± 5.1%; *P* < 0.05) was no longer present in stressed rats (rostral 63.7 ± 5.7%, caudal 72.4 ± 2.0%; *P* > 0.05). Scale bar = 10 μm; *n* = 5. ^*^P < 0.05.

To study segregation of neurotransmitters in sympathetic ganglia, we explored expression and co-occurrence of VAChT and GAD67 in ganglionic varicosities originating from the cell bodies of SPN that, in turn, were labeled for ChAT and GAD67. Although both isoforms of GAD, GAD65 and GAD67, contribute to GABA synthesis, we chose GAD67 because GAD65 has been found in mouse SCG (Ito et al., 2005), while in rat SCG, GAD67 has been used (Ito et al., 2007; Elinos et al., 2016). In fact, we tried antibodies directed to each isoforms and we got better labeling to GAD67. The other and more important reason to choose GAD67 was for its major role in regulation of synaptic plasticity (Lau and Murthy, 2012). We retrograde-labeled SPN cell bodies with FG, we found 15–20 cells per animal, all were ChAT positive and some of them (4–5) were positive to GAD67, these GAD67 immunostained cells were always positive to ChAT (Figures 2A,B). In the SCG of control rats approximately half of GAD67-containing varicosities lack VAChT, and there is a caudo-rostral gradient of segregation of VAChT-GAD67 (Elinos et al., 2016). We found that in the SCG of cold stressed rats segregation of GAD67-VAChT increased from 50.1 ± 3.6% (control) to 67.8 ± 4.4% (*P* < 0.05; Figure 3), and also found that the caudo-rostral gradient of segregation of VAChT-GAD67 was not present (rostral 63.7 ± 5.7%, caudal 72.4 ± 2.0%; *P* > 0.05; Figure 3B). Change in segregation induced by stress is mainly due to a considerable and significant increase of segregation in the rostral area, which overcomes any regional intraganglionic differences.

To confirm that the immunolabeled varicosities studied have a preganglionic origin, we denervated the SCG of four rats by transecting the preganglionic sympathetic trunk 3–5 mm caudal to the ganglia. After 7 days of denervation, we found a complete lack of GAD67- and VAChT-containing varicosities confirming their preganglionic origin (Supplementary Figure S1).

#### Expression of GAD67 and VAChT Did Not Change in the SCG of Cold Stressed Rats

**Figure 4:**
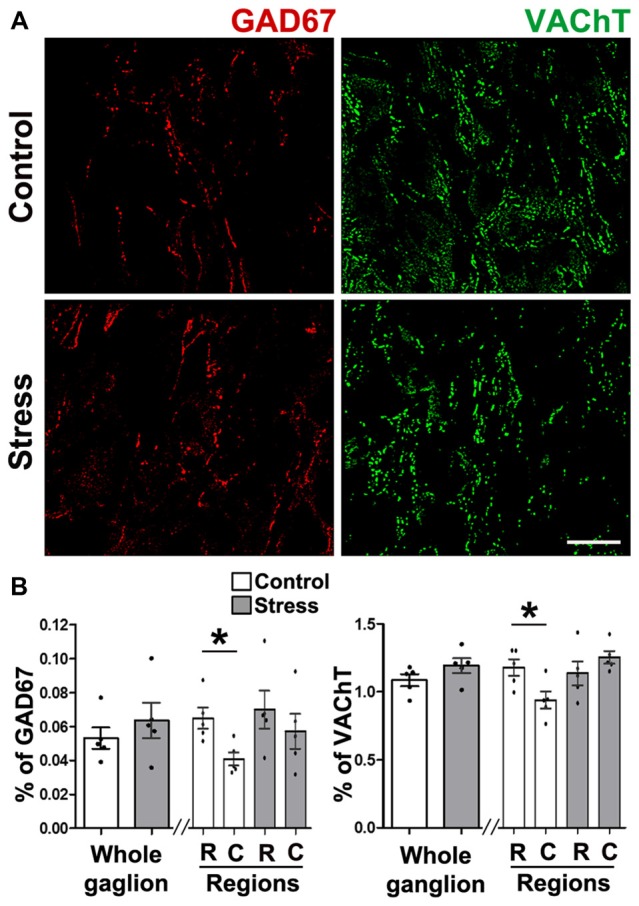
Expression of GAD67 and VAChT in SCG was not modified by cold-stress. **(A)** GAD67 showed similar presence in control and stressed rats (0.053 ± 0.003% in control and 0.064 ± 0.005% in stress; *P* > 0.05). GAD67 showed a rostro-caudal gradient in control rats (0.07 ± 0.01% rostral vs. 0.04 ± 0.01% caudal;* P* < 0.05) that was not present in stressed rats (0.07 ± 0.01% rostral vs. 0.06 ± 0.01% caudal; *P* > 0.05). **(B)** VAChT in SCG from control and stressed rats showed no differences in whole ganglia (1.09 ± 0.05% in control and 1.23 ± 0.03% in stress; *P* > 0.05), or in ganglionic regions in control rats (1.20 ± 0.07% rostral vs. 0.93 ± 0.08% caudal; *P* < 0.05) and in stress (1.20 ± 0.07% rostral vs. 1.28 ± 0.05% caudal; *P* > 0.05). Scale bar = 25 μm; *n* = 5. ^*^P < 0.05.

To explain the changes in segregation in cold stressed rats, we quantified GAD67 and VAChT, and found that content of both markers was not affected by cold stress. However, neurotransmitter segregation increased (see above) suggesting that they were redistributed. The density of GAD67 was 0.053 ± 0.003% in controls vs. 0.064 ± 0.005% in stressed animals (*P* > 0.05; Figures 4A,B), whereas VAChT density in controls was 1.09 ± 0.05%, which was not different to animals subjected to stress (1.23 ± 0.03%, *P* > 0.05; Figures 4A,B). Unlike controls, ganglion of rats subjected to cold stress did not show a rostro-caudal gradient of GAD67 expression (Figures 4A,B; see Elinos et al., 2016), instead they showed uniform expression of the enzyme throughout the ganglion (0.07 ± 0.01% in rostral and 0.06 ± 0.01% in caudal; *P* > 0.05; Figures 4A,B). VAChT-IR showed regional differences in controls but not in animals subjected to stress.

### Hypertension Model

#### Segregation of ACh-GABA Was Similar in SHR and WKy

We confirmed the presence of sympathetic hyperactivity in SHR by assessing the basal sympathetic ganglionic transmission. We measured compound action potentials (CAP) in the postganglionic nerve evoked by supramaximal stimulation applied in the preganglionic nerve. We found that CAP amplitude in ganglia from SHR was higher than from WKy rats, (Vmax was 2.7 ± 0.2 mV in SHR vs. 1.7 ± 0.1 mV in WKy; *P* < 0.001).

**Figure 5:**
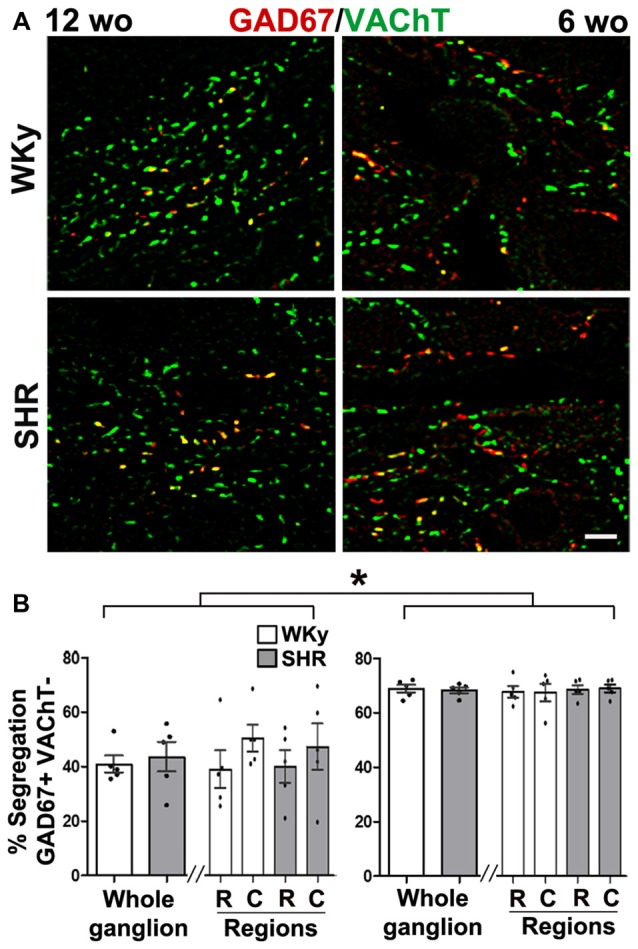
Hypertension did not modify segregation of GAD67/VAChT in SCG from SHR of adult or young rats. However, segregation was larger in young than in adult SHR rats. **(A)** Merged images showing the co-occurrence (yellow) of two labels: GAD67 (red) and VAChT (green) in 12- and 6- wo SHR and WKy rats. **(B)** Regional distribution of the level of segregation of GAD67/VAChT in 12- and 6-wo SHR and Wistar Kyoto (WKy) rats. Percent of ACh and GABA segregation in the whole ganglion was larger in young than in adult SHR and WKy rats (68.3 ± 1.1% at 6 wo and 43.5 ± 5.4% at 12 wo; *P* < 0.001) for SHR and (68.9 ± 1.4% at 6 wo and 40.8 ± 3.1% at 12 wo; *P* < 0.001) for WKy. This larger segregation in young than in adult rats was also found at intraganglionic regional level, in SHR rostral (68.6 ± 1.6% at 6 wo and 40.0 ± 6.0% at 12 wo; *P* < 0.001) caudal (69.0 ± 1.5% at 6 wo and 47.2 ± 8.5% at 12 wo; *P* < 0.01); while in WKy rostral (67.8 ± 2.1% at 6 wo and 40.0 ± 6.9% at 12 wo; *P* < 0.001) caudal (67.6 ± 3.2% at 6 wo and 50.3 ± 4.9% at 12 wo; *P* < 0.001). Scale bar = 10 μm; *n* = 5 for all groups. ^*^P < 0.01.

Like in control rats, GAD67 and ChAT co-occurred in preganglionic cell bodies of SHR animals at the two age groups tested, i.e., 12- and 6-wo animals (Figures 2C,D). We found a clear segregation of VAChT and GAD67 in the preganglionic varicosities within the SCG in SHR and normotensive WKy rats. The level of segregation of VAChT-GAD67 did not vary between SHR and WKy rats regardless of the age analyzed. Thus, in adult 12-wo SHR, we found that 43.5 ± 5.4% of GAD67-containing preganglionic varicosities lacked VAChT, whereas in WKy rats the level of segregation was 40.8 ± 3.1% (*P* > 0.05; Figures 5A,B). In young animals, we detected same levels of segregation, 68.3 ± 1.1% in SHR and 68.9 ± 1.4% in Wky (*P* > 0.05; Figures 5A,B). Contrary to our findings in control animals, we did not find a caudo-rostral gradient of segregation for VAChT-GAD67 in SHR or WKy rats (Figure 5B).

Denervation of ganglia, removed all VAChT and GAD67-containing varicosities, indicating a preganglionic origin (Supplementary Figure S1).

#### GABA Expression Was Augmented in SCG of Adult and Young SHR. ACh Increased Only in Young SHR Rats

**Figure 6:**
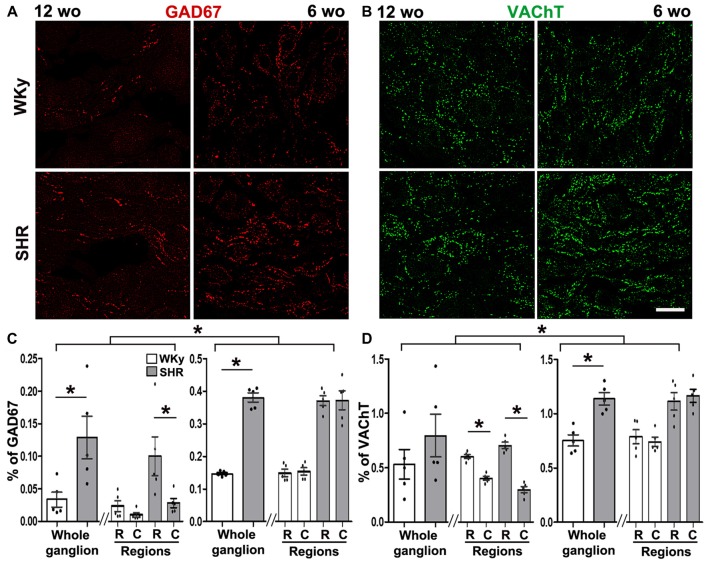
Hypertension increased GAD67 expression in 12- and 6-wo rats and VAChT expression in 6-wo animals. **(A)** Immunostaining of SCG for GAD67 at 12- and 6-wo SHR and WKy rats. **(B)** GAD67-containing varicose fibers increased in adult and young SHR rats in the whole ganglion (0.12 ± 0.03% in SHR and 0.03 ± 0.01% in WKy at 12 wo; *P* < 0.01; in young animals: 0.37 ± 0.01% in SHR and 0.140 ± 0.003% in WKy; *P* < 0.001). At 12 wo in SHR rostral expression of GAD67 was larger than in caudal region (rostral 0.10 ± 0.02%; caudal 0. 020 ± 0.007%; *P* < 0.05). **(C)** Immunostaining of SCG for VAChT in 12 and 6 wo in SHR and WKy rats. **(D)** Although there were no changes in VAChT expression between SHR and WKY at 12 wo (0.79 ± 0.19%, in SHR; 0.52 ± 0.13%, in WKy, *P* > 0.05), VAChT expression was greater in SHR at 6 wo (1.13 ± 0.05% in SHR and 0.75 ± 0.05%, in WKy; *P* < 0.01). At 12 wo, there was a rostro-caudal gradient in VAChT expression in both strands SHR (rostral 0.70 ± 0.02%; caudal 0.29 ± 0.02%; *P* < 0.001); WKy (rostral 0.60 ± 0.01%; caudal 0.39 ± 0.01%; *P* < 0.001). At 6 wo, there was no regional distribution in VAChT expression SHR (1.11 ± 0.08% rostral and 1.16 ± 0.06% caudal, *P* > 0.05); WKy (0.78 ± 0.06% rostral and 0.74 ± 0.04% caudal, *P* > 0.05). Scale bar = 25 μm; *n* = 5 for all groups. ^*^P < 0.05.

We found that GAD67 increased in the SCG of adult and young SHR. In adult rats, we detected 4–5 times more GAD67-containing varicosities in SHR than in WKy, thus, GAD67 density was 0.13 ± 0.03% in SHR vs. 0.03 ± 0.01% in WKy (*P* < 0.01; Figures 6A,B). In the same way, in young animals, GAD67 was detected in 0.40 ± 0.01% of varicosities in SHR and in 0.150 ± 0.004% of WKy rats (*P* < 0.001; Figures 6A,B). Regarding regionalization of GAD67 expression, it is worth emphasizing that while adult SHR GAD67 showed a rostro-caudal gradient (0.10 ± 0.03%, rostral vs. 0.03 ± 0.01% caudal; *P* < 0.05; Figure 6B), adult WKy showed no gradient (0.02 ± 0.01%, rostral and 0.010 ± 0.003% caudal; *P* > 0.05; Figure 6B). In turn, in young SHR and WKy rats this rostro-caudal gradient was not present. VAChT expression was similar in adult SHR and WKy rats, and showed a rostro-caudal distribution in both normotensive and hypertensive conditions (0.70 ± 0.02%, rostral and 0.30 ± 0.02% caudal in SHR; *P* < 0.001; and 0.60 ± 0.01%; rostral and 0.40 ± 0.01% caudal in WKy; *P* < 0.001; Figure 6D). In young animals, in contrast to adult rats, expression of VAChT was higher in SHR (1.14 ± 0.06% compared with WKy rats 0.75 ± 0.05%; *P* < 0.01; Figures 6C,D). The rostro-caudal distribution of VAChT-IR was absent in young animals (Figure 6D).

#### Segregation of VAChT and GABA Was Greater in Young Animals

It is noteworthy that, although, segregation did not vary between SHR and WKy rats, as stated above, it varied depending on the age. In SCG from young 6-wo SHR the percentage of GAD67-containing varicosities lacked VAChT, 68.3 ± 1.1% was significantly greater than in 12-wo adult SHR animals, 43.5 ± 5.4% (*P* < 0.001; Figures 5A,B).

## Discussion

Data presented here show that under both sympathetic overfunction conditions, cold stress and hypertension, the expression and segregation of neurotransmitters in the rat SCG varied differentially depending on the type of overfunction. In cold stressed rats, segregation of ACh and GABA increased without changes in their expression levels. The opposite was found in hypertensive animals, sympathetic overactivity coursed with a higher presence of GABA, but without any changes in the segregation level of ACh and GABA. Additionally, we unexpectedly found that young animals (6-wo) showed a higher level of ACh-GABA segregation than adult ones (12-wo).

We previously demonstrated that in sympathetic ganglia, both *in vitro* and* in vivo*, segregation of neurotransmitters is a plastic phenomenon, which is modulated by neurotrophic factors and neurotrophins. Thus, addition of ciliary neurotrophic factor (CNTF) increases the degree of segregation of NE and NPY in varicosities of sympathetic ganglionic neurons co-cultured with cardiomyocytes (Vega et al., 2010). Furthermore, the neurotrophin, nerve growth factor (NGF) changes the segregation of ACh and methionine-enkephalin (mENK) in preganglionic sympathetic neuronal varicosities *in vivo* (Vega et al., 2016). Considering these evidences, we explored whether plastic changes in the segregation of neurotransmitters can also occur in physio-pathologic conditions, like stress and hypertension, conditions that course with augmented sympathetic activity.

It is known that stress involves the activation of central sympathetic nuclei and peripheral structures. Generally, the grade of contribution of sympathetic ganglia in stress is assessed by the level of catecholamines released into ganglionic neuronal targets (Kvetnansky et al., 2013). However, since the entire sympathetic axis is involved in stress it is expected that other sympathetic structures, like preganglionic neurons, may be compromised as well. In fact, it has been proposed that SPN increase ACh release during stress (Ulus and Wurtman, 1979). Our study focused on characterizing the cellular distribution of two SPN neurotransmitters, ACh and GABA, in the SCG of cold stressed rats. We found that there was no change in presynaptic ACh or GABA content, but a likely redistribution of them, which increased the segregation of ACh from GABA-containing varicosities. This finding suggests that sympathetic hyperactivity occurring in stress is directly correlated with the level of segregation. Thus, we postulate that an increase in segregation of ACh and GABA may lead to a stronger transmission in sympathetic ganglia, resulting from less GABA inhibitory modulation. In line with this hypothesis in a recent work where we explored possible intraganglionic regional differences in the level of ACh and GABA segregation, we found larger segregation levels in the caudal region of SCG, which correlates with more potent sympathetic transmission (Elinos et al., 2016). As we suggested in that work, it seems that inhibitory modulation of GABA on cholinergic transmission is less effective when GABA is released alone from separate boutons than when both neurotransmitters are co-localized and co-released from the same bouton. In other systems some functional implications of segregation have been reported, for example in microcultures of single dopaminergic neurons, dopamine and glutamate are segregated in two types of varicosities: a dopaminergic varicosity, which is involved in volume transmission, and a glutamatergic one that mediates rapid excitatory synaptic transmission (Sulzer et al., 1998). In the retina, it has been proposed that ACh and GABA, segregated and released from different presynaptic endings of single processes of starburst amacrine cells, are used to process independently different visual tasks (Lee et al., 2010).

Considering the differential level of ACh and GABA expression, and the segregation detected in the rostral and caudal regions of the SCG of control rats, we explored whether effects of cold-stress on expression and segregation of ACh and GABA are regionally distributed. We found that cold-stress increased segregation preferentially in the rostral region. This increased segregation would reduce GABA modulatory inhibition. This results in strengthening of ganglionic transmission, which may contribute to the stress-induced sympathetic overfunction.

Regarding hypertension, we found that the segregation of neurotransmitters in sympathetic ganglia of SHR was not modified indicating that ganglionic segregation is not affected by the sympathetic overfunction underlying hypertension. Instead, we detected a significant increase in GABA presence. We also detected stronger synaptic transmission in SHR which can explain sympathetic overfunction in this pathological condition. Therefore, the enhancement of GABA expression would seem contradictory to this assumption, as one would expect a reduction in the presence and function of GABA-inhibition. A plausible explanation for this apparent contradiction is that GABA increased in an attempt to counteract the already established enhancement of sympathetic transmission coursing with hypertension.

According to the contradictory findings in segregation and expression levels of ACh and GABA detected in the two conditions of sympathetic overactivity, stress and hypertension, and considering that cold-stress is a short-lasting condition of sympathetic hyperfunction, whereas hypertension represents a long-lasting overactivity, it is possible to speculate that in stress the SNS responds by increasing segregation of ACh and GABA, which results in an enhancement of sympathetic activity. While in hypertension by increasing GABA content the SNS tries to overcome the already established sympathetic overactivity.

In view of our previous report of regional differences in the strength of ganglionic transmission, and the heterogeneous distribution of GABA and GABA-A receptors in the SCG of adult (10–12-wo) Wistar rats (Elinos et al., 2016), we investigated possible differences in expression of GABA and ACh in rostral and caudal SCG regions of SHR. We found a rostro-caudal gradient in GAD67 and VAChT expression in the SCG of adult SHR that contrasted with young 6-wo animals, where this gradient was not present. The regional changes in neurotransmitter presence in ganglia could be related to the different target organs innervated by rostral and caudal neurons.

Notwithstanding that in hypertension segregation of VAChT and GAD67 did not vary, we discovered an age-dependent level of segregation both in SHR and WKy rats, segregation was larger at 6-wo. We also found higher GAD67 and VAChT expression in 6-wo animals, in contrast to adult 12-wo SHR, where only GAD67 increased. In other regions, like the spinal cord, a developmental sequence of GABA excitatory/inhibitory effects has been reported, e.g., in spinal cord this switch occurs during the first postnatal week (Ben-Ari, 2014). In ganglia, we know that at 6-wo, GABA already shows an inhibitory effect (unpublished observation). Data of more segregation in young animals suggest that at early age neurotransmitters involved in co-release are largely located in separate axon terminals, and as the animals get older neurons modify expression and intracellular distribution of their neurotransmitters favoring a greater co-storage of mediators in the same boutons. Finally, the higher level of segregation at early age would favor independent release of GABA, which may exert other functions than synaptic cholinergic inhibition, like modulation of development, as it was suggested elsewhere (Wolff et al., 1987). As the animals get older the developmental functions of GABA are no longer required, therefore the number of GABA-containing varicosities lacking of VAChT would reduce, resulting in less segregation.

According to the data presented here we propose that in the SNS of the rat, the content and intracellular distribution of transmitters might contribute in setting the activity strength of this system. Segregation and expression of neurotransmitters can be differentially modulated in physio-pathological conditions that course with sympathetic hyperactivity, like stress and hypertension. In stress, sympathetic neurons respond by increasing segregation of ACh and GABA, which might result in enhancement of sympathetic activity that characterizes stress. While in hypertension, by increasing GABA content, neurons try to overcome the already established sympathetic overactivity. Moreover, we detected that segregation and expression of transmitters was different between sympathetic neurons of young and adult subjects.

## Author Contributions

MM and FC conceived and designed the research. CM-J, FM, MZR and JP performed the experiments. CM-J prepared figures. CM-J, MM and FC analyzed and interpreted the results. MM and FC edited and revised the manuscript. All authors approved the final version of the manuscript and agree to be accountable for all aspects of the work.

## Conflict of Interest Statement

The authors declare that the research was conducted in the absence of any commercial or financial relationships that could be construed as a potential conflict of interest.

## Supplementary Material

FIGURE S1 Denervation of SCG removed practically all immunostaining for GAD67 and VAChT. Micrographs of GAD67 **(A)** and VAChT **(B)** immunostaining in intact and denervated SCG.
