# Supplementary figures and images for: Sympathetic Hyperactivity and Age Affect Segregation and Expression of Neurotransmitters
Source: Front Cell Neurosci. 2018 Nov 13;12:411. doi: 10.3389/fncel.2018.00411 (PMC6243098; doi:10.3389/fncel.2018.00411)

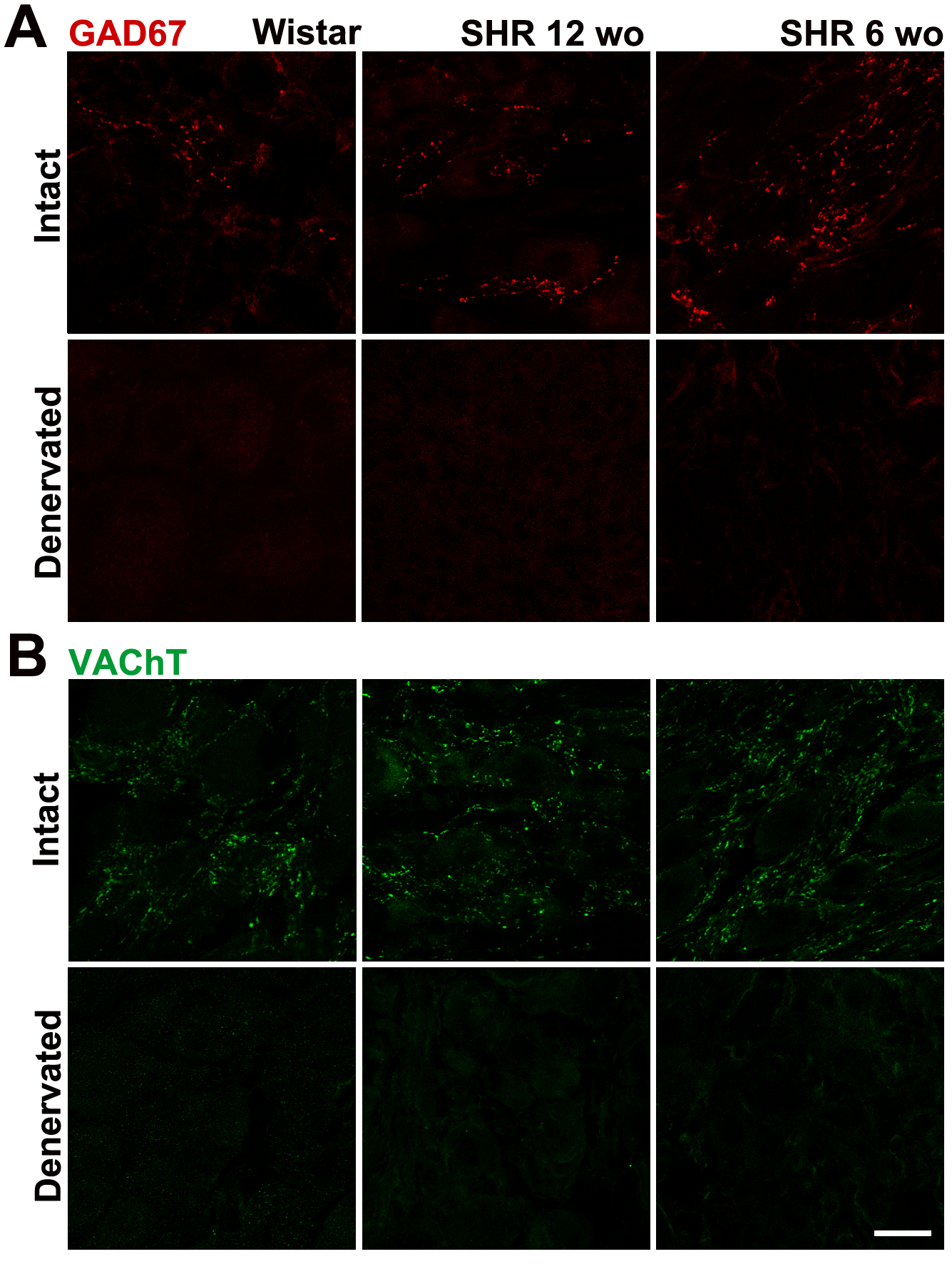

Supplement: FIGURE S1 — Denervation of SCG removed practically all immunostaining for GAD67 and VAChT. Micrographs of GAD67 (A) and VAChT (B) immunostaining in intact and denervated SCG. [file Image_1.TIF]
